# Involution of Breast Lobules, Mammographic Breast Density and Prognosis Among Tamoxifen-Treated Estrogen Receptor-Positive Breast Cancer Patients

**DOI:** 10.3390/jcm8111868

**Published:** 2019-11-04

**Authors:** Maeve Mullooly, Sarah J. Nyante, Ruth M. Pfeiffer, Renata Cora, Donna Butcher, Lawrence Sternberg, Erin J. Aiello Bowles, Shaoqi Fan, Jonine D. Figueroa, Sheila Weinmann, Robert N. Hoover, Louise A. Brinton, Amy Berrington de Gonzalez, Andrew Glass, Mark E. Sherman, Gretchen L. Gierach

**Affiliations:** 1 Division of Population Health Sciences, Royal College of Surgeons in Ireland, D02 YN77 Dublin, Ireland; 2 Division of Cancer Epidemiology and Genetics, National Cancer Institute, Bethesda, MD 20892, USA; pfeiffer@mail.nih.gov (R.M.P.); shaoqi.fan@nih.gov (S.F.); hooverr@exchange.nih.gov (R.N.H.); brintonl@exchange.nih.gov (L.A.B.); berringtona@mail.nih.gov (A.B.d.G.); gierachg@mail.nih.gov (G.L.G.); 3 Department of Radiology and Lineberger Comprehensive Cancer Center, University of North Carolina at Chapel Hill, Chapel Hill, NC 27599, USA; sarah_nyante@med.unc.edu; 4 Independent Contractor, CT(ASCP), MB(ASCP), Stamford, CT 06901, USA; renata.cora@nih.gov; 5 Pathology-Histotechnology Laboratory, Frederick National Laboratory for Cancer Research sponsored by the National Cancer Institute, Frederick, MD 21702, USA; butcherdo@mail.nih.gov (D.B.); sternberglr@mail.nih.gov (L.S.); 6 Kaiser Permanente Washington Health Research Institute, Seattle, WA 98101, USA; Erin.A.Bowles@kp.org; 7 Usher Institute of Population Health Sciences and Informatics, CRUK Edinburgh Centre, Medical School, The University of Edinburgh, Teviot Place, Edinburgh EH8 9AG, UK; jonine.figueroa@ed.ac.uk; 8 Kaiser Permanente Northwest Center for Health Research, Portland, OR 97227, USA; Sheila.Weinmann@kpchr.org (S.W.); andy_5241@msn.com (A.G.); 9 Mayo Clinic, Jacksonville, FL 32224, USA; Sherman.Mark@mayo.edu

**Keywords:** breast cancer, mammographic breast density, involution, terminal duct lobular unit

## Abstract

Mammographic breast density (MD) reflects breast fibroglandular content. Its decline following adjuvant tamoxifen treated, estrogen receptor (ER)-positive breast cancer has been associated with improved outcomes. Breast cancers arise from structures termed lobules, and lower MD is associated with increased age-related lobule involution. We assessed whether pre-treatment involution influenced associations between MD decline and risk of breast cancer-specific death. ER-positive tamoxifen treated patients diagnosed at Kaiser Permanente Northwest (1990–2008) were defined as cases who died of breast cancer (*n* = 54) and matched controls (remained alive over similar follow-up; *n* = 180). Lobule involution was assessed by examining terminal duct lobular units (TDLUs) in benign tissues surrounding cancers as TDLU count/mm^2^, median span and acini count/TDLU. MD (%) was measured in the unaffected breast at baseline (median 6-months before) and follow-up (median 12-months after tamoxifen initiation). TDLU measures and baseline MD were positively associated among controls (*p* < 0.05). In multivariable regression models, MD decline (≥10%) was associated with reduced risk of breast cancer-specific death before (odds ratio (OR): 0.41, 95% CI: 0.18–0.92) and after (OR: 0.41, 95% CI: 0.18–0.94) adjustment for TDLU count/mm^2^, TDLU span (OR: 0.34, 95% CI: 0.14–0.84), and acini count/TDLU (OR: 0.33, 95% CI: 0.13–0.81). MD decline following adjuvant tamoxifen is associated with reduced risk of breast cancer-specific death, irrespective of pre-treatment lobule involution.

## 1. Introduction

Approximately 80% of breast cancers in the United States (US) are estrogen receptor (ER)-positive and incidence rates are increasing [1]. Although treatment with the selective estrogen receptor modulator (SERM) tamoxifen dramatically reduces mortality related to these cancers [2], fatal recurrences occur at a rate of 1%–2% per year over a long-term follow-up of 15 years. Accordingly, identifying women who are most likely to respond to tamoxifen treatment, at early stages of their treatment, would improve clinical management.

High mammographic breast density (MD), which reflects the percentage of breast parenchyma comprised of fibroglandular tissue, is associated with elevated breast cancer risk overall and for ER-positive breast cancers [3,4]. We and others showed that women who experience MD decline one year following initiation of adjuvant tamoxifen treatment have reduced risk of breast cancer-specific mortality compared to women who do not experience a decline [5,6,7,8]. There are multiple mechanisms proposed to modify the response to tamoxifen therapy, often leading to intrinsic or acquired resistance [9]. Molecular characteristics of breast tumors that have been shown to influence tamoxifen efficacy include crosstalk of growth factor signaling with ER signaling pathways as well as upregulation of phosphoinositide-3-kinase/AKT signaling downstream of ERα [10]. In addition, pharmacologic mechanisms related to drug activity also influence response to tamoxifen therapy. Specifically, inter-individual differences in the capacity to convert the parent drug to its more active metabolites may influence tamoxifen effectiveness [9]. It has been hypothesized that a decline in MD may represent the activity of such metabolites. Further, at the histological level, treatment with tamoxifen has shown to reduce stromal density [11] and epithelial mass [12] in rodents. Taken together, these studies suggest that MD decline may represent a biosensor of treatment response.

The fibroglandular component of the breast is comprised of non-fatty stroma and terminal duct lobular units (TDLUs), which include the majority of at risk epithelial cells [13]. TDLUs involute with aging and are replaced by fatty tissue and collagen, processes that can be influenced by reproductive factors including parity and menopause [14]. TDLUs have been described as a principal source and location for breast cancer, and reduced levels of TDLU involution is associated with increased risk of developing breast cancer among women diagnosed with benign breast disease [15,16,17,18]. Additionally, failure of lobule involution to progress among women who underwent benign biopsies at two time points was linked to increased breast cancer risk compared with women who experience age-related progressive lobule involution over time [19].

Both higher MD and reduced TDLU involution are global measures of breast cancer risk. As MD decline is also associated with lower risk of breast cancer following tamoxifen therapy in the prevention setting [20], it biologically plausible that the pre-treatment baseline level of lobule involution may influence the associations observed between these two risk factors. Indeed, prior studies have shown that there are moderate correlations between high MD and reduced TDLU involution [21,22].

Upon a diagnosis of breast cancer, little is known about the role of TDLU involution in tumor progression and prognosis [23,24]. We hypothesize that the biological mechanisms underlying improved breast cancer outcomes associated with MD decline may be influenced by the degree of TDLU involution at diagnosis. Therefore, in this study we assess whether levels of TDLU involution in benign tissues adjacent to breast cancers influence the relationship between MD decline and improved breast cancer-specific survival among ER-positive breast cancer patients treated with tamoxifen in a general community health care plan.

## 2. Methods

### 2.1. Study Population

Women in this study were part of a previously conducted case-control study designed to evaluate the prognostic significance of MD change, as described in detail elsewhere [6]. That prior study included 349 patients who were selected from a cohort of 2315 Kaiser Permanente Northwest health care plan (KPNW; Portland, OR, USA) members diagnosed with ER-positive primary invasive breast cancer of localized or regional stage, between 1990 and 2008 and who were treated with adjuvant tamoxifen therapy. Briefly, cases were patients who died of breast cancer during follow-up, through 31 December 2010; vital status was ascertained from the KPNW tumor registry. The control population included breast cancer patients who were alive at follow-up or had died from other causes but that had not died from breast cancer as of the last tumor registry follow-up during the study period (31 December 2010). Control patients were matched to each case on age at diagnosis, tumor stage and year of diagnosis, using the categories outlined in more detail below, and were sampled to have at least as much follow-up time as the matched case patient. Approval to carry out this study was obtained by the Special Studies Institutional Review Board of the National Cancer Institute and the Institutional Review Board of KPNW.

Covariate data were obtained from electronic and paper medical records as previously described [6]. Data were categorized for analysis and included age at diagnosis, body mass index (BMI) at diagnosis, race, smoking status, tumor stage at diagnosis, calendar year of diagnosis, progesterone receptor (PR) status, tumor size and tumor grade. Prescription records and treatment data were obtained from KPNW databases, including duration of tamoxifen use and menopausal hormone therapy use, [6].

### 2.2. Breast Tissue Collection

**Figure 1:**
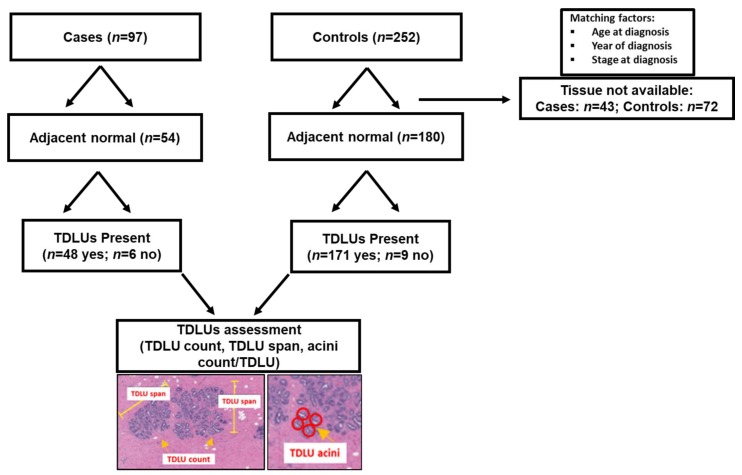
Overview of the study population of breast cancer cases and controls with benign breast tissue suitable for terminal duct lobular unit (TDLU) assessment in this study and representative examples of TDLU measures.

Available archived hematoxylin and eosin (H&E)-stained tissue sections from breast surgeries related to the primary breast cancer diagnosis were reviewed by our study pathologist (MES) to assess tumor pathology and the availability of adjacent non-tumor tissue sections suitable for analysis of TDLU involution (Figure 1).

### 2.3. TDLU Histologic Assessment

Digital whole-slide images of one representative H&E-stained breast tissue section from each patient were generated at 20× magnification (Leica Aperio AT2, Vista, CA, USA) and compiled for web-based review and digital measurement/annotation (Leica Aperio Imagescope software v12.3; Vista, CA, USA). Three independent standardized measurements of TDLU involution were assessed [25] by the ASCP certified cyto-technician (RC). First, the number of normal TDLUs was visually counted and standardized for tissue area (TDLU count/mm^2^). Second, the average TDLU span, i.e., the average size in microns of the longest axis of up to 10 TDLUs, was determined [24]. Finally, the number of acini per each TDLU was determined using a semi-automated image analysis tool [26]. Median values for each patient were used as summary measures of TDLU span and acini count (i.e., median TDLU span and median acini count/TDLU). To ensure reproducibility, a repeat assessment was carried out by the study cyto-technician (RC) in a masked fashion on a subset of 50 H&E-stained tissue sections that were selected using a stratified random sample approach across all age categories to ensure a wide range of TDLU involution levels. Results from this reliability assessment showed strong intra-observer agreement for each of the three TDLU measures, with intraclass correlation coefficients (ICCs) as follows: TDLU counts = 0.85; median TDLU span = 0.81 and median acini count/TDLU = 0.92.

### 2.4. Mammographic Breast Density (MD) Assessment

Assessment of MD was carried out as previously described [6]. Briefly, baseline and follow-up craniocaudal film mammographic views from the contralateral breast were obtained for each woman: a median of 6 months prior to the initiation of tamoxifen initiation (baseline) and a median of 10.8 months post-tamoxifen initiation (follow-up). The median time between baseline mammogram and follow-up mammograms in this analytical population was 15.6 months (for case patients 15.4 months and for control patients 15.6 months). Films were digitized using an Array Corporation 2095 Laser Film Digitizer (Roden, the Netherlands; optical density = 4.0). Using Cumulus software [27], a single reader (EAB) assessed mammographic images for absolute dense area (cm^2^) and total breast area (cm^2^). High reproducibility of MD assessment was confirmed as previously described [6]. MD measurements assessed in this analysis included absolute dense area (cm^2^), total breast area (cm^2^), non-dense area (cm^2^) (total breast area–absolute dense area), percent density ((dense area/total breast area) × 100) and percent MD density change (percent density at follow-up−percent density at baseline).

### 2.5. Statistical Analysis

Descriptive characteristics were compared between case patients and control patients using chi-square Fisher exact, and Wilcoxon rank sum tests as appropriate. Univariate relationships between patient and tumor characteristics and TDLU measurements were assessed using the Kruskal–Wallis test. Relationships between TDLU measurements were evaluated using Spearman’s rank correlation coefficients. To determine relationships between tertiles of TDLU measures and continuous MD measures analysis of covariance (ANCOVA) models were used, adjusting for age, stage, year of diagnosis and BMI. MD measures were square-root transformed to better approximate normal distributions. For ease of interpretation, back transformed least square means, with calculated corresponding 95% confidence intervals, are presented by tertiles of TDLU measures. Tertiles of TDLU measures were defined according to distributions of measures among control patients.

We undertook two approaches to investigate the influence of TDLU metrics on the relationship between MD decline and risk of breast cancer death: adjustment and stratification. In the former approach, odds ratios (OR) and 95% confidence intervals (CI) for the association between MD decline of 10% or greater and breast cancer-specific death were estimated using unconditional logistic regression, initially adjusting for matching factors only (age, stage and year of diagnosis) and case follow-up time, and then additionally separately adjusting for each TDLU metric. Unconditional logistic regression was used to retain statistical power given that not all cases and controls had tissue available for TDLU analysis. The cut-point of 10% for MD decline was selected based on previously published data showing that a decline of this magnitude has been consistently associated with improved breast cancer outcomes in this and other study populations [5]. In the second approach, we evaluated the association between MD decline and breast cancer-specific death in unconditional logistic regression models stratified by the median of each TDLU metric based on the distribution among control patients. All statistical analyses were carried out using SAS 9.3 (SAS Institute Inc., Cary, NC, USA). *p*-values of <0.05 were considered statistically significant and all tests were two-tailed.

## 3. Results

### 3.1. Characteristics of Study Population

Clinicopathological characteristics of the final study population are shown in Table 1. Compared with control patients, case patients had tumors that were less frequently well-differentiated or PR-positive. Case patients were more likely to be smokers and had a significantly shorter duration of tamoxifen therapy (Table 1).

Descriptive statistics comparing the demographic and clinical characteristics of the original case-control patient population [6] with the final current study population with available adjacent normal tissue blocks for TDLU assessment, overall and by case-control status, are shown in Supplementary Table S1A,B, respectively. Overall 54 cases (56% of the original case-set within the case-control study [6]) and 180 control (71% of the original control set from the case-control study [6]) patients had tissue available for analysis. Compared with patients with available benign tissues, those whose tissues were unavailable were more likely to have died from breast cancer (Supplementary Table S1A). Similar case-control differences were observed for patients with and without available benign tissues (Supplementary Table S1B).

### 3.2. Relationships between Participant Characteristics and TDLU Measurements

TDLUs were observed in 95% of cases and 89% of controls. TDLU count/mm^2^ was modestly correlated with TDLU span and acini count/TDLU, while TDLU span and acini count/TDLU were strongly correlated with each other (Supplementary Table S2). The relationships between patient characteristics and TDLU measurements are shown in Table 2. All TDLU measurements declined with increasing age, irrespective of case-control status. TDLU count/mm^2^ was also inversely associated with BMI, though only significantly associated among control patients (*p* = 0.04). No statistically significant associations were observed between any of the tumor characteristics investigated and TDLU count/mm^2^ or span. Among control patients, acini count/TDLU was significantly higher among patients with regional/distant disease compared with those with localized disease (*p* = 0.03).

### 3.3. Association between MD and Measures of TDLU Involution

Relationships were observed between TDLU and MD measures among control patients, for whom positive associations were observed with baseline % MD (TDLU count/mm^2^: *p* = 0.03; TDLU span: *p* = 0.03 and median acini count/TDLU: *p* < 0.01; Table 3). Similar patterns of association with baseline % MD were observed for case patients, although associations with TDLU count and acini/TDLU were not statistically significant and only TDLU span showed a statistically significant positive association with baseline % MD (*p* < 0.01). Positive associations were also observed for relationships of TDLU metrics with baseline absolute dense area, though statistically significant associations were only observed among case patients for TDLU span (*p* < 0.01) and acini count/TDLU (*p* < 0.01). Inverse associations between TDLU measurements and baseline non-dense area were largely restricted to control patients. With respect to TDLU associations with follow-up % MD (post-tamoxifen), patterns of association appeared similar to those observed for baseline % MD for both cases and controls; however, strengths of associations were attenuated for all TDLU measures (Table 3).

### 3.4. Influence of TDLU Measurements on the Relationship between MD Decline and Reduced Risk of Breast Cancer-Specific Death among ER-positive Patients Treated with Tamoxifen

The relationship between a 10% or greater reduction in % MD (between baseline and follow-up mammograms) and reduced risk of breast cancer-specific death among this analytic population of patients with slides available for lobule involution evaluation was statistically significant (OR: 0.41 and 95% CI: 0.18, 0.92; Table 4). This finding was consistent with our previously reported association in all case-control patients (i.e., including those both with and without available benign slides for analysis (OR: 0.42 and 95% CI: 0.22, 0.80 [6])). Similar associations were observed after additional adjustment for TDLU count/mm^2^ (OR: 0.41 and 95% CI: 0.18, 0.94), TDLU span (OR: 0.34 and 95% CI: 0.14, 0.84) or median acini count/TDLU (OR: 0.33 and 95% CI: 0.13, 0.81) and when all the TDLU measures were included in the model (OR: 0.33 and 95%CI 0.13, 0.81; Table 4). In analyses stratified by the median of each TDLU metric, the patterns of association between density reduction and reduced risk of breast cancer-specific death persisted, though confidence limits were wide likely due to sparse numbers in TDLU subgroups (Supplementary Table S3). Although there was some suggestion of a stronger protective effect of MD decline among those with higher baseline TDLU counts, a test for interaction between TDLU count/mm^2^ and change in percent MD was null (*p*-interaction = 0.52). In sensitivity analysis, additional adjustment for duration of tamoxifen use and smoking did not substantially alter the observed associations (data not shown).

## 4. Discussion

Decline in MD has been linked to improved outcomes among women with ER-positive breast cancers treated with tamoxifen, as previously reported in this cohort [6]. Given that higher MD and less TDLU involution are correlated, yet independent measures of breast cancer risk, we investigated whether measurement of pre-diagnostic lobular involution modifies the prognostic significance of MD decline among patients receiving adjuvant tamoxifen. Our results show that reduced levels of involution, assessed as higher TDLU counts/mm^2^, larger TDLU span, or a greater number of acini counts/TDLU, did not significantly alter the relationship between MD decline and reduced risk of breast cancer-specific death among this population of ER-positive breast cancer patients treated with tamoxifen. We identified TDLUs in most of the breast cancer specimens, irrespective of patient case or control status. Consistent with prior reports [28], we also found relationships between TDLU metrics and breast cancer risk factors, which followed expected patterns, including inverse associations with age, and a positive relationship with baseline MD.

Lobule involution, a histologic measure, has been hypothesized to be reflected radiologically in MD, a breast cancer risk marker for which the underlying biology is not fully elucidated [28]. A recent analysis that examined TDLUs among 1115 women who had serial biopsies over time from the Mayo Benign Breast Disease (BBD) Cohort, highlighted the importance of persistent TDLUs over time as a breast cancer biomarker [19]. In that study, the authors showed that no progression of the involution process, i.e., persistent TDLUs, between the initial and subsequent biopsies was associated with a larger increase in risk of breast cancer development compared with women who experienced progression of lobular involution between biopsies [19]. Thus, while progression of involution over time may be protective in women, delayed involution (persistent TDLUs) may contribute to breast cancer development, supporting the importance of understanding the relevance of the degree of lobular involution at the time of and subsequent to breast cancer diagnosis.

Compared to studies that have examined the relationships between TDLU involution and breast cancer risk, less is known about the role of involution at diagnosis. Herein, we report for the first time that levels of involution at diagnosis did not significantly alter the relationship between MD decline and reduced risk of breast cancer-specific death among ER-positive breast cancer patients treated with tamoxifen. Our results raise interesting mechanistic questions about the observed association between density decline and improved breast cancer outcomes with tamoxifen. The original observation of this association was reported in the IBIS (International Breast Cancer Intervention Study)-I chemoprevention trial in which preventive therapy with tamoxifen was associated with a lower risk of incident breast cancer, suggesting a possible direct effect on at-risk breast epithelium [20]. Recent data using novel volumetric MD measurements suggest that treatment with aromatase inhibitors may also produce declines in MD [29], although the prognostic impact remains unclear in the prevention and adjuvant settings. Given that breast cancer deaths are attributable almost exclusively to metastatic disease, it is unlikely that the mechanisms mediating the prognostic effect of tamoxifen-related MD decline are fully related to effects on the breast. Previously, we suggested that declines in density might represent a biosensor of tamoxifen effectiveness, potentially related to bioavailability of potent tamoxifen metabolites [5]. How tamoxifen treatment alters breast fibroglandular pathology, as reflected in MD decline at the radiological level, is not well understood and requires further studies examining breast tissue both pre- and post-tamoxifen treatment, to fully investigate histological biomarkers of MD change in this setting. Analysis of murine models that demonstrate tamoxifen associated declines in mammary fibroglandular tissue composition may uncover molecular mechanisms underlying these associations [11]. Additional studies investigating relationships between MD decline and tamoxifen metabolism, and future window of opportunity clinical studies with pre- and post-treatment tissues, will be crucial in understanding these relationships.

Although associations of TDLU involution with age and MD were as expected in our study, we observed a higher proportion of pathology specimens containing TDLUs than prior reports [24,30,31,32]. In contrast to prior reports [31], our population differed in that it was nearly exclusively white and included the highest proportion of obese women. Further, our analyses used sections from tissue blocks adjacent to the tumor, whereas the proximity of tissue sections in relation to cancers may have differed in other reports. Limitations of this study included the sample size, which led to limited statistical power, particularly for stratified analysis of MD change. An additional limitation includes the absence of information on reproductive factors including menopausal status and parity, two factors that are both related to MD and TDLU involution [14]. Further, we were unable to examine changes in TDLU involution over time, following tamoxifen treatment. The strengths of this study include the use of benign breast tissue sections to examine lobular involution, contributing to the characterization of histology of benign tissue surrounding invasive breast cancer. A further strength was the use of reproducible standardized metrics to assess TDLU involution, along with quantitative assessment of MD and MD decline measurements. An additional strength includes the integration of histological data from archival tissue with the radiological data from mammograms into this retrospective cohort of breast cancer survivors, which aims to identify factors associated with breast cancer prognosis.

In conclusion, our findings suggest that TDLU involution levels at breast cancer diagnosis were unrelated to tamoxifen effectiveness in the adjuvant setting. MD decline following adjuvant tamoxifen initiation was associated with reduced risk of breast cancer-specific death, and thus acting as potential biosensor of tamoxifen effectiveness, irrespective of pre-tamoxifen treatment TDLU involution levels. Future studies to examine change in TDLU involution over time following tamoxifen treatment in the adjuvant and breast cancer prevention setting may provide useful information with regard to predicting favorable responses.

## Figures and Tables

**Table 1:** Selected patient and tumor characteristics among ER-positive breast cancer patients treated with tamoxifen with available adjacent normal tissue blocks for TDLU assessment.

	All Patients		Case Patients		Control Patients		Comparison of Case Patients and Control Patients
	(*n* = 234)		(*n* = 54)		(*n* = 180)		
Characteristic	*n*	%	*n*	%	*n*	%	*p* Value ^$^
**Age at diagnosis (years)**							0.20
<50	59	25.2	14	25.9	45	25.0	
50–59	65	27.8	10	18.5	55	30.6	
>59	110	47.0	30	55.6	80	44.4	
							
**Race**							0.58*
White	229	97.9	175	97.2	54	100.0	
Non-white	4	1.7	4	2.2	0	0.0	
Missing	1	0.4	1	0.6	0	0.0	
							
**Smoking status at baseline**							0.02
Never	128	54.7	22	40.7	106	58.9	
Ever	106	45.3	32	59.3	74	41.1	
							
**BMI at diagnosis (kg/m^2^)**							0.16
<25	72	30.8	11	20.4	61	33.9	
25 to <30	64	27.4	16	29.6	48	26.7	
≥30	67	28.6	19	35.2	48	26.7	
Missing	31	13.2	8	14.8	23	12.8	
							
**Hormone therapy use at baseline**							0.79
Non-user	92	39.3	22	40.7	70	38.9	
Current user	104	44.4	22	40.7	82	45.6	
Former user	38	16.2	10	18.5	28	15.6	
							
**SEER summary stage at diagnosis**							0.90
Localized	98	41.9	23	42.6	75	41.7	
Regional/distant/unknown	136	58.1	31	57.4	105	58.3	
							
**Year of diagnosis**							0.16
1990–1996	93	39.7	23	42.6	70	38.9	
1997–2000	79	33.8	22	40.7	57	31.7	
2001–2008	62	26.5	9	16.7	53	29.4	
							
**Progesterone receptor**							**0.02**
Negative	48	20.5	17	31.5	31	17.2	
Positive	185	79.1	37	68.5	148	82.2	
Missing	1	0.4	0	0.0	1	0.6	
							
**Tumor size (mm)**							0.08
≤2	141	60.3	26	48.1	115	63.9	
>2	84	35.9	24	44.4	60	33.3	
Missing	9	3.8	4	7.4	5	2.8	
							
**Tumor differentiation**							<0.01 *
Well differentiated	44	18.8	1	1.9	43	23.9	
Moderately differentiated	123	52.6	32	59.3	91	50.6	
Poorly differentiated	54	23.1	17	31.5	37	20.6	
Missing	13	5.6	4	7.4	9	5.0	
							
**Duration of tamoxifen use (months)**							
Mean (SD)	50.4 (19.9)		42.6 (20.3)		52.7 (19.3)		**<0.01 #**

^$^ Chi-Squared analysis; * Fisher exact test; # Wilcoxon rank sum test; *p*-Values <0.05 are highlighted in bold font.

**Table 2:** Relationship between patient and tumor characteristics and TDLU measurements among ER-positive case and control breast cancer patients treated with tamoxifen.

Case patients (*n* = 54)										Control patients (*n* = 180)								
	TDLU Count/100 mm^2^			TDLU Span^&^ (Microns)			Acini Count/TDLU^&^			TDLU Count/100 mm^2^			TDLU Span^&^ (Microns)			Acini Count/TDLU^&^		
	Median	IQR	*p* Value	Median	IQR	*p* Value	Median	IQR	*p* Value	Median	IQR	*p* Value	Median	IQR	*p* Value	Median	IQR	*p* Value
**All patients**	5.9	1.3–10.0		416	284.0–665.5		21	14.0–42.3		7.0	2.7–16.3		465	312.5–631.5		24	11.5–46.5	
																		
**Age at diagnosis (years)**			**<0.01**			**0.01**			**0.01**			**<0.01**			**<0.01**			**<0.01**
<50	14.7	8.7–27.8		677	409.8–928.5		43	30.0–65.0		11.4	5.5–21.7		594	477.0–768.5		40	24.3–56.8	
50–59	7.5	3.6–22.9		406	331.5–606.5		23	12.5–49.0		12.5	4.6–25.5		487	368.0–655.0		28	15.0–51.0	
>59	3.5	1.0–7.5		348	245.0–465.0		18	12.0–22.5		3.9	2.0–9.1		342	271.0–496.0		14	8.5–25.5	
																		
**Race**												**0.03**			0.09			0.09
White	5.9	1.3–10.0	−	416	284.0–665.5	−	21	14.0–42.3	−	6.8	2.7–15.0		453	307.0–628.5		24	11.5–46.0	
Non-white	0.0	−		0	−		0	−		27.8	13.9–44.0		650	515.8–929.3		50	33.5–61.8	
																		
**Smoking status at baseline**			0.16			0.61			0.51			0.18			0.06			0.14
Never	3.8	1.0–9.5		399	269.5–606.5		19	12.0–40.0		8.3	2.7–18.6		512	328.5–687.3		25	11.5–53.5	
Ever	8.3	2.5–10.6		421	331.5–671.0		22	14.5–43.5		6.2	2.8–11.3		425	299.5–575.5		20	10.5–40.5	
Missing																		
																		
**BMI at diagnosis (kg/m^2^)**			0.57			0.27			0.19			**0.04**			0.72			0.33
<25	7.1	2.3–11.1		380	227.5–448.0		16	8.0–27.0		8.0	4.6–19.5		518	307.0–675.5		25	14.0–53.5	
25 to <30	4.7	1.4–9.2		353	245.0–494.5		16	12.0–21.0		7.4	2.8–15.6		422	298.0–614.5		20	10.5–51.0	
≥30	3.9	0.9–9.9		432	308.8–648.3		23	16.0–44.8		6.1	2.1–10.6		477	323.0–655.0		28	11.5–40.5	
Missing																		
																		
**Hormone therapy use at baseline**			0.21			0.88			0.76			0.70			0.19			0.34
Non-user	3.3	0.6–9.5		399	239.0–729.0		22	8.0–40.0		7.6	3.1–18.6		474	334.0–675.5		25	11.5–51.5	
Current user	6.7	3.6–10.0		418	341.5–606.5		20	16.0–41.0		7.0	2.6–15.0		477	317.0–640.5		24	13.5–50.0	
Former user	9.2	1.3–21.8		450	286.0–671.0		31	14.0–53.0		5.3	2.4–18.5		358	271.0–605.5		15	10.5–34.0	
																		
**SEER summary stage at diagnosis**			0.97			0.75			0.77			0.16			0.05			0.03
Localized	5.8	0.9–21.8		380	237.3–686.0		20	11.0–40.5		5.6	2.5–14.8		440	284.8–614.3		18	10.5–45.5	
Regional/distant/unknown	6.0	1.5–9.7		422	325.3–610.0		21	14.0–44.8		8.4	3.2–17.4		487	344.5–658.5		28	14.5–51.0	
																		
**Year of Diagnosis**			0.67			0.21			0.40			0.62			0.09			0.25
1990–1996	5.8	1.6–22.9		495	348.0–688.0		23	16.0–63.5		6.9	3.1–18.6		425	297.0–587.0		20	11.3–39.3	
1997–2000	5.1	1.0–9.7		399	245.0–606.5		19	12.0–36.0		6.5	2.5–14.7		477	334.0–655.0		24	13.5–51.5	
2001–2008	7.1	1.0–10.0		320	275.8–666.3		17	13.3–29.3		7.7	4.2–16.3		552	349.0–683.0		35	13.0–46.0	
																		
**Progesterone Receptor**			0.50			0.76			0.92			0.64			0.91			0.91
Negative	5.4	1.3–9.7		424	331.5–497.0		19	14.5–27.5		8.2	3.4–24.1		468	302.0–640.50		26	15.0–41.5	
Positive	7.1	1.6–11.1		411	269.5–684.0		22	12.0–49.0		7.0	2.7–14.9		466	318.0–627.3		24	11.5–50.5	
Missing																		
																		
**Tumor Size (cm)**			0.86			0.21			0.24			0.61			0.07			0.06
≤2	6.7	2.7–21.2		421	282.0–683.0		19	14.5–46.0		6.7	3.1–14.8		449	298.0–615.5		23	11.0–45.0	
>2	4.7	0.7–9.6		399	269.5–671.0		22	12.0–41.0		8.3	2.6–18.7		542	346.0–655.0		29	17.0–51.5	
Missing																		
**Tumor differentiation**			0.22			0.55			0.55			0.56			0.58			0.80
Well differentiated	0.0	−		0	−		0	−		8.0	4.6–21.4		446	304.8–596.3		23	12.8–43.3	
Moderately differentiated	7.3	3.1–14.6		399	245.0–671.0		19	12.0–46.0		7.3	2.5–15.0		518	303.3–676.8		25	11.5–51.5	
Poorly differentiated	3.6	1.0–10.0		436	320.0–648.8		22	15.3–42.3		6.1	2.8–17.4		431	334.0–589.0		24	14.0–39.5	
Missing																		
**Duration of tamoxifen use, mo**			0.24			0.52			0.17			0.46			0.06			0.16
≤52	5.1	1.0–21.2		411	2860–729.0		23	16.0–49.0		9.4	3.2-19.9		558	396.0–745.0		29	16.0–54.5	
53 to 61	1.5	1.0–4.2		354	245.0–448.0		15	9.5–21.5		7.3	2.9–13.3		429	304.0–606.5		24	11.5–45.5	
>61	8.7	6.0–9.7		458	319.0–513.5		20	12.0–31.0		5.2	2.5–18.8		465	297.0–614.5		20	11.5–41.0	

IQR: interquartile range; TDLU: terminal duct lobular unit and &: among patients with TDLUs observed. *p*-values were estimated using Kruskal–Wallis tests and were computed excluding the missing categories. *p*-values <0.05 are highlighted in bold font. Tertiles based on the distribution among control patients.

**Table 3:** Relationship between TDLU measurements and baseline and follow-up MD measurements among ER-positive case and control patients treated with tamoxifen.

TDLU measure	Baseline Density (%)			Baseline Dense Area (cm^2^)		Baseline Non-Dense Area (cm^2^)		Follow-Up Density (%)		Breast Density Change (%)	
	*N*	Mean	(95% CI)	Mean	(95% CI)	Mean	(95% CI)	Mean	(95% CI)	Mean	(95% CI)
**Case patients**											
**TDLU count/100 mm^2^**											
0	6	26.0	(18.8, 33.2)	43.2	(30.5, 56.0)	121.1	(94.6, 147.7)	17.1	(11.5, 22.7)	−8.5	(−15.3, −1.7)
0–≤4.7	18	17.7	(14.2, 21.3)	31.5	(24.9, 38.0)	151.8	(134.0, 169.6)	18.4	(14.9, 21.9)	0.2	(−3.9, 4.2)
>4.7–≤13	19	26.1	(21.5, 30.6)	30.7	(24.0, 37.5)	100.8	(85.6, 116.0)	22.8	(18.8, 26.9)	−3.0	(−7.3, 1.3)
>13	11	32.0	(23.1, 40.8)	55.6	(39.6, 71.6)	112.2	(84.0, 140.4)	26.3	(18.6, 34.0)	−4.6	(−12.1, 2.9)
*p*-value for trend		0.15		0.61		0.11		0.07		0.75	
**Median TDLU span (microns)**											
0–≤352	18	17.8	(14.4, 21.2)	26.5	(21.2, 31.7)	133.7	(114.6, 152.9)	19.3	(15.8, 22.7)	1.5	(−2.4, 5.4)
>352–≤578	15	26.3	(22.0, 30.6)	35.4	(29.1, 41.8)	109.3	(91.1, 127.5)	23.1	(19.1, 27.1)	−3.6	(−7.7, 0.5)
>578	15	33.6	(26.7, 40.5)	57.1	(45.7, 68.6)	114.1	(87.9, 140.3)	25.8	(19.9, 31.8)	−6.5	(−12.3, -0.7)
*p*-value for trend		**<0.01**		**<0.01**		0.31		0.19		**0.02**	
**Median acini count per TDLU**											
0–≤15	15	20.5	(16.2, 24.8)	27.4	(21.3, 33.5)	121.6	(100.8, 142.4)	20.2	(16.2, 24.2)	−0.5	(−5.0, 4.1)
>15–≤36.5	19	22.7	(18.6, 26.7)	33.3	(27.3, 39.2)	122.0	(103.4, 140.6)	21.8	(18.1, 25.5)	−1.3	(−5.4, 2.8)
>36.5	14	33.1	(25.7, 40.5)	57.2	(45.4, 69.0)	116.2	(88.9, 143.6)	25.7	(19.7, 31.8)	−6.1	(−12.3, 0.0)
*p*-value for trend		0.08		**<0.01**		0.85		0.35		0.21	
											
**Control patients**											
**TDLU count/100 mm^2^**											
0	9	26.7	(19.7, 33.6)	30.8	(20.6, 41.1)	103.7	(80.9, 126.5)	22.5	(16.4, 28.6)	−6.2	(−12.6, 0.2)
0–≤4.7	57	23.9	(21.1, 26.7)	33.5	(28.9, 38.0)	110.8	(100.8, 120.9)	19.6	(17.2, 22.0)	−5.2	(−7.9, −2.5)
>4.7–≤13	57	30.5	(27.5, 33.5)	40.6	(35.8, 45.4)	97.8	(88.8, 106.8)	25.9	(23.3, 28.6)	−4.5	(−7.1, −1.9)
>13	57	31.5	(28.3, 34.8)	39.5	(34.4, 44.5)	84.9	(75.9, 93.9)	22.4	(19.8, 25.1)	−9.4	(−12.2, −6.6)
*p*-value for trend		**0.03**		0.15		**0.02**		0.36		0.09	
**Median TDLU span (microns)**											
0–≤352	57	25.7	(22.6, 28.8)	34.3	(29.4, 39.3)	105.5	(95.5, 115.5)	20.6	(17.9, 23.3)	−6.0	(−8.9, −3.1)
>352–≤578	57	28.3	(25.4, 31.4)	37.3	(32.5, 42.1)	94.2	(85.4, 102.9)	23.5	(20.8, 26.1)	−4.6	(−7.3, −1.9)
>578	57	32.8	(29.6, 35.9)	41.6	(36.6, 46.5)	87.7	(79.4, 96.0)	24.7	(22.0, 27.3)	−8.5	(−11.2, −5.9)
*p*-value for trend		**0.03**		0.17		0.07		0.17		0.18	
**Median acini count per TDLU**											
0–≤15	57	24.1	(21.3, 27.0)	32.8	(28.2, 37.4)	109.7	(100.0, 119.4)	20.2	(17.6, 22.7)	−4.8	(−7.6, −2.0)
>15–≤36.5	56	29.1	(26.1, 32.1)	38.1	(33.3, 42.9)	93.7	(85.2, 102.3)	23.3	(20.7, 25.9)	−5.9	(−8.6, 3.2)
>36.5	57	34.1	(30.8, 37.4)	42.7	(37.6, 47.9)	83.8	(75.6, 92.1)	25.5	(22.7, 28.3)	−8.6	(−11.4, −5.9)
*p*-value for trend		**<0.01**		0.06		**<0.01**		0.06		0.07	

Breast density change was defined as (follow-up % density-baseline % density) such that negative value indicates a decrease in % density over follow-up. Analysis of covariance (ANCOVA) models were used, adjusting for age, stage, year of diagnosis and BMI. MD measures were square-root transformed to better approximate normal distributions. For ease of interpretation, back transformed least square means, with calculated corresponding 95% confidence intervals, are presented by tertiles of TDLU measures. Tertiles of TDLU measures were defined according to distributions of measures among control patients. *p*-values <0.05 are highlighted in bold font.

**Table 4:** Relationships between MD decline* and breast cancer-specific death before and after adjustment for TDLU measurements among ER-positive patients treated with tamoxifen.

	OR (95% CI)
Base model ^$^	0.41 (0.18, 0.92)
Base model adjusted for TDLU count/mm^2^	0.41 (0.18, 0.94)
Base model adjusted for TDLU span ^&^	0.34 (0.14, 0.84)
Base model adjusted for acini count/TDLU ^&^	0.33 (0.13, 0.81)
Base model adjusted for TDLU count, span and acini/TDLU	0.33 (0.13, 0.81)

CI: confidence interval, MD: mammographic density, OR: odds ratio, TDLU: terminal duct lobular unit. * Mammographic density decline defined as a ≥ 10% decrease between baseline and follow-up mammograms: ≥10% (*n* = 9 cases, *n* = 61 controls); <10% (reference group: *n* = 45 cases, *n* = 119 controls). ^$^ Base model adjusted for age, stage, year of diagnosis, follow-up time (all as categorical variables). ^&^ among women with observed TDLUs.

## Data Availability

The data that support these findings are not publicly available because they contain information that could compromise research participant privacy and confidentiality. The authors will make the data available upon reasonable request and with permission of the Kaiser Permanente Center for Health Research in Portland, Oregon.

## Supplementary Materials

The following are available online at https://www.mdpi.com/2077-0383/8/11/1868/s1, Supplementary Table S1A: Selected patient and tumor characteristics among tamoxifen treated ER-positive breast cancer patients with benign tissue available for analysis compared to ER-positive breast cancer patients with no tissue available for analysis in this study (Sensitivity analysis). Supplementary Table S1B: Comparison of selected patient and tumor characteristics among ER-positive breast cancer case or control patients with tissue and available for analysis in this study versus ER-positive breast cancer case and control patients with no tissue available for analysis (Sensitivity analysis). Supplementary Table S2: Relationships between TDLU measurements (TDLU counts/100mm^2^, TDLU span and acini counts/TDLU). Supplementary Table S3: Relationships between MD decline and breast cancer-specific death stratified by baseline median TDLUs measurements.

## Abbreviations

BMI	Body mass index
ER	Estrogen receptor
H&E	Hematoxylin and eosin
KPNW	Kaiser Permanente Northwest
MD	Mammographic breast density
OR	Odds ratio
TDLU	Terminal duct lobular unit
SERM	Selective estrogen receptor modulator
