# Supplementary material for: Involution of Breast Lobules, Mammographic Breast Density and Prognosis Among Tamoxifen-Treated Estrogen Receptor-Positive Breast Cancer Patients
Source: J Clin Med. 2019 Nov 4;8(11):1868. doi: 10.3390/jcm8111868 (PMC6912285; doi:10.3390/jcm8111868)
Supplement: Supplementary file 1 [file jcm-08-01868-s001.pdf]

Supplementary Table 1A. Selected patient and tumor characteristics among ER-positive breast cancer patients with benign tissue available for analysis compared to ER-positive breast cancer patients with no tissue available for analysis in this study (sensitivity analysis).

	**Total**		**Patients with tissue available**				
	**(*N* = 349)**		**Yes (*N* = 234)**		**No (*N* = 115)**		***P* value** [$]
Characteristic	*N*	%	*N*	%	*N*	%	
Age at diagnosis (years)							0.33
<50	88	25.2	59	25.2	29	25.2	
50–59	89	25.5	65	27.8	24	20.9	
>59	172	49.3	110	47.0	62	53.9	
Race							0.45 *
White	340	97.4	229	97.9	111	96.5	
Non-white	8	2.3	4	1.7	4	3.5	
Missing	1	0.3	1	0.4	0	0.0	
Smoking status at baseline							0.18
Never	199	57.0	128	54.7	71	61.7	
Ever	149	42.7	106	45.3	43	37.4	
Missing	1	0.3	0	0.0	1	0.9	
BMI at diagnosis (kg/m2)							0.13
<25	98	28.1	72	30.8	26	22.6	
25 to <30	105	30.1	64	27.4	41	35.7	
>=30	107	30.7	67	28.6	40	34.8	
Missing	39	11.2	31	13.2	8	7.0	
Hormone therapy use at baseline							0.10
Non-user	151	43.3	92	39.3	59	51.3	
Current user	144	41.3	104	44.4	40	34.8	
Former user	54	15.5	38	16.2	16	13.9	
SEER summary stage at diagnosis							0.29
Localized	153	43.8	98	41.9	55	47.8	
Regional/distant/unknown	196	56.2	136	58.1	60	52.2	
Year of Diagnosis							0.89
1990-1996	141	40.4	93	39.7	48	41.7	
1997-2000	115	33.0	79	33.8	36	31.3	
2001-2008	93	26.6	62	26.5	31	27.0	
Progesterone Receptor							0.63
Negative	69	19.8	48	20.5	21	18.3	
Positive	278	79.7	185	79.1	93	80.9	
Missing	2	0.6	1	0.4	1	0.9	
Tumor Size (mm)							0.73
≤ 2	209	59.9	141	60.3	68	59.1	
> 2	128	36.7	84	35.9	44	38.3	
Missing	12	3.4	9	3.8	3	2.6	
Tumor differentiation							0.47
Well differentiated	68	19.5	44	18.8	24	20.9	
Moderately differentiated	176	50.4	123	52.6	53	46.1	
Poorly differentiated	86	24.6	54	23.1	32	27.8	

Missing	19	5.4	13	5.6	6	5.2	
Duration of tamoxifen use, months							0.73 #
Mean (Std Error)	49.6 (1.1)		50.4 (1.3)		47.9 (1.8)		
Breast Cancer Death							**<0.01**
No	252	72.2	180	76.9	72	62.6	
Yes	97	27.8	54	23.1	43	37.4	

$ Chi-Squared analysis; * Fisher exact test; *P* values were computed excluding missing categories; # Wilcoxon rank sum test; *P*-values <0.05 are highlighted in bold font.

Supplementary Table 1B. Comparison of selected patient and tumor characteristics among ER-positive breast cancer case or control patients with tissue and available for analysis in this study versus ER-positive breast cancer case and control patients with no tissue available for analysis (sensitivity analysis).

	Case patients							Control patients						
	Total	Case tissue available						Total	Control tissue available					
	(*N* = 97)	Yes (*N* = 54)		No (*N* = 43)		Comparison of case patients with versus without tissue		(*N* = 252)	Yes (*N* = 180)		No (*N* = 72)		Comparison of control patients with versus without tissue	Comparison of cases and controls without tissue
**Variable**	***N***	***N***	**%**	***N***	**%**	***P* value$**	***N***	***N***	**%**	***N***	**%**	***P* value$**	***P* value$**
**Age at diagnosis (years)**						0.75						0.19	0.37
**<50**	28	14	25.9	14	32.6		60	45	25.0	15	20.8		
**50-59**	18	10	18.5	8	18.6		71	55	30.6	16	22.2		
**>59**	51	30	55.6	21	48.8		121	80	44.4	41	56.9		
**Race**						0.44*						0.41*	1.00
**White**	96	54	100.0	42	97.7		244	175	97.2	69	95.8		
**Non-white**	1	0	0.0	1	2.3		7	4	2.2	3	4.2		
**Missing**	0	0	0.0	0	0.0		1	1	0.6	0	0.0		
**Smoking status at baseline**						0.31						0.14	0.06
**Never**	44	22	40.7	22	51.2		155	106	58.9	49	68.1		
**Ever**	53	32	59.3	21	48.8		96	74	41.1	22	30.6		
**Missing**	0	0	0.0	0	0.0		1	0	0.0	1	1.4		
**BMI at diagnosis (kg/m2)**						0.86						**0.04**	0.67
**<25**	22	11	20.4	11	25.6		76	61	33.9	15	20.8		
**25 to <30**	29	16	29.6	13	30.2		76	48	26.7	28	38.9		
**>=30**	33	19	35.2	14	32.6		74	48	26.7	26	36.1		
**Missing**	13	8	14.8	5	11.6		26	23	12.8	3	4.2		
**Hormone therapy use at baseline**						0.20						0.47	0.28
**Non-user**	47	22	40.7	25	58.1		104	70	38.9	34	47.2		
**Current user**	33	22	40.7	11	25.6		111	82	45.6	29	40.3		

**Former user**	17	10	18.5	7	16.3		37	28	15.6	9	12.5		
**SEER summary stage at diagnosis**						0.94						0.16	0.32
**Localized**	41	23	42.6	18	41.9		112	75	41.7	37	51.4		
**Regional/distant/ unknown**	56	31	57.4	25	58.1		140	105	58.3	35	48.6		
**Year of Diagnosis**						0.16						0.64	0.57
**1990-1996**	40	23	42.6	17	39.5		101	70	38.9	31	43.1		
**1997-2000**	34	22	40.7	12	27.9		81	57	31.7	24	33.3		
**2001-2008**	23	9	16.7	14	32.6		70	53	29.4	17	23.6		
**Progesterone Receptor**						0.37						0.73	0.30
**Negative**	27	17	31.5	10	23.3		42	31	17.2	11	15.3		
**Positive**	70	37	68.5	33	76.7		208	148	82.2	60	83.3		
**Missing**	0	0	0.0	0	0.0		2	1	0.6	1	1.4		
**Tumor Size (mm)**						0.59						0.62	**0.02**
**≤ 2**	45	26	48.1	19	44.2		164	115	63.9	49	68.1		
**> 2**	46	24	44.4	22	51.2		82	60	33.3	22	30.6		
**Missing**	6	4	7.4	2	4.7		6	5	2.8	1	1.4		
**Tumor differentiation**						0.65*						0.28	**<0.01** *
**Well differentiated**	1	1	1.9	0	0.0		67	43	23.9	24	33.3		
**Moderately differentiated**	55	32	59.3	23	53.5		121	91	50.6	30	41.7		
**Poorly differentiated**	34	17	31.5	17	39.5		52	37	20.6	15	20.8		
**Missing**	7	4	7.4	3	7.0		12	9	5.0	3	4.2		
**Duration of tamoxifen use, mo**						0.47#						0.32#	**<0.01** #
**Mean (Std Error)**	40.9 (2.0)	42.6 (2.8)		38.9 (2.8)			52.9 (1.2)	52.7 (1.4)		53.4 (2.1)			
**Breast Cancer Death**						n/a						n/a	**<0.01** *
**No**	0	0	0.0	0	0.0		252	180	100.0	72	100.0		
**Yes**	97	54	100.0	43	100.0		0	0	0.0	0	0.0		

$ Chi-Squared analysis * Fisher exact test *P* values were computed excluding missing categories. #Wilcoxon rank sum test *P*-values <0.05 are highlighted in bold font.

Supplementary Table 2. Relationships between TDLU measurements (TDLU counts, TDLU span and acini counts/TDLU).

	TDLU count/100 mm^2		TDLU span$^{\&}$ (microns)	
-	r	*P* value	r	*P* value
TDLU count/100 mm^2	1.00	-	-	-
TDLU span & (microns)	0.52	<0.0001	1.00	-
Acini count/TDLU &	0.48	<0.0001	0.90	<0.0001

TDLU: terminal duct lobular unit & Among patients with TDLUs observed P-values were estimated using Spearmans rank correlation coefficients. P-values <0.05 highlighted in bold font.

Supplementary Table 3: Relationships between MD-decline and breast cancer-specific death stratified by median baseline TDLUs measurements.

	Low (< median)			High (≥ median)		
	**Case**	**Control**	**Base model OR (95% CI)**	**Case**	**Control**	**Base model OR (95% CI)**
TDLU count/mm^2						
MD change <10%	25	69	Ref.	20	50	Ref.
MD change ≥10%	4	21	0.62 (0.18, 2.06)	5	40	0.27 (0.09, 0.83)
TDLU span[1]						
MD change <10%	28	66	Ref.	13	47	Ref.
MD change ≥10%	2	20	0.22 (0.05, 1.06)	5	38	0.52 (0.16, 1.68)
TDLU acini counts[1]						
MD change <10%	27	66	Ref.	14	46	Ref.
MD change ≥10%	2	20	0.23 (0.05, 1.13)	5	38	0.43 (0.13, 1.40)

OR = odds ratio, CI = confidence interval; Reference group for all analysis included women with breast density change <10%; Base model adjusted for age, stage, year of diagnosis, follow-up time (all as categorical variables); 1among women with observed TDLUs. Median cut off for TDLU span (low/high) was 465 (median cut-off based on controls with TDLUs). Median cut off for TDLU counts (low/high) was 7.04 (median cut-off based on distribution including 0's). Median cut off for TDLU acini was 24 (analyzer acini accessment: median cut-off based on controls with TDLUs).
